# *catena*-Poly[[bis­(pyrazine-2-carbox­amide)mercury(II)]-di-μ-chlorido]

**DOI:** 10.1107/S1600536810003879

**Published:** 2010-02-06

**Authors:** Alireza Azhdari Tehrani, Bahareh Mir Mohammad Sadegh, Hamid Reza Khavasi

**Affiliations:** a Department of Chemistry, Shahid Beheshti University, G.C., Evin, Tehran 1983963113, Iran

## Abstract

In the polymeric title compound, [HgCl_2_(C_5_H_5_N_3_O)_2_]_*n*_, the Hg^II^ atom (site symmetry 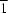) adopts a distorted *trans*-HgN_2_Cl_4_ octa­hedral coordination geometry. In the crystal, adjacent mercury ions are bridged by pairs of chloride ions, generating infinite [100] chains, and N—H⋯O and N—H⋯(N,N) hydrogen bonds help to consolidate the packing.

## Related literature

For related structures, see: Cati & Stoeckli-Evans (2004 ▶); Hausmann & Brooker (2004 ▶); Mir Mohammad Sadegh *et al.* (2010 ▶); Miyazaki *et al.* (2007 ▶).
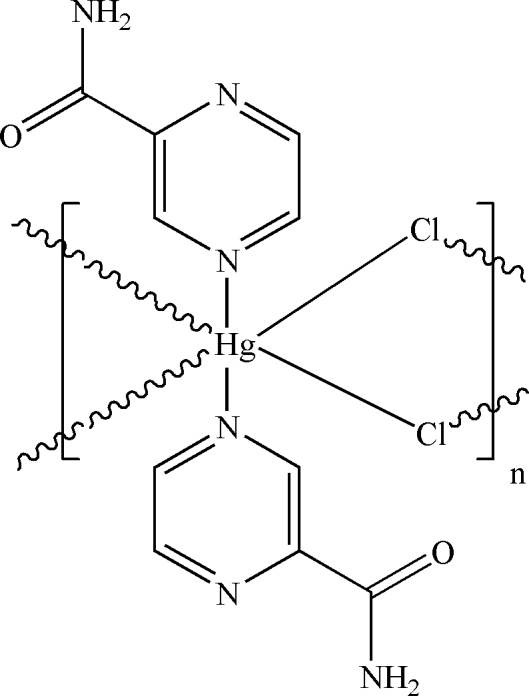


## Experimental

### 

#### Crystal data

[HgCl_2_(C_5_H_5_N_3_O)_2_]
*M* *_r_* = 517.73
Triclinic, 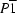

*a* = 3.8451 (8) Å
*b* = 6.4170 (13) Å
*c* = 14.854 (3) Å
α = 101.14 (3)°
β = 92.53 (3)°
γ = 94.69 (3)°
*V* = 357.73 (13) Å^3^
*Z* = 1
Mo *K*α radiation
μ = 11.14 mm^−1^
*T* = 298 K
0.48 × 0.15 × 0.06 mm

#### Data collection

Stoe IPDS II diffractometer
Absorption correction: numerical [optically, by *X-RED* and *XSHAPE* (Stoe & Cie, 2005 ▶)] *T*_min_ = 0.150, *T*_max_ = 0.515
4201 measured reflections
1887 independent reflections
1880 reflections with *I* > 2σ(*I*)
*R*_int_ = 0.096

#### Refinement

*R*[*F* ^2^ > 2σ(*F* ^2^)] = 0.054
*wR*(*F* ^2^) = 0.144
*S* = 1.08
1887 reflections
97 parameters
H-atom parameters constrained
Δρ_max_ = 3.25 e Å^−3^
Δρ_min_ = −3.75 e Å^−3^

### 

Data collection: *X-AREA* (Stoe & Cie, 2005 ▶); cell refinement: *X-AREA*; data reduction: *X-AREA*; program(s) used to solve structure: *SHELXTL* (Sheldrick, 2008 ▶); program(s) used to refine structure: *SHELXTL*; molecular graphics: *ORTEP-3* (Farrugia, 1997 ▶); software used to prepare material for publication: *WinGX* (Farrugia, 1999 ▶).

## Supplementary Material

Crystal structure: contains datablocks global, I. DOI: 10.1107/S1600536810003879/hb5301sup1.cif

Structure factors: contains datablocks I. DOI: 10.1107/S1600536810003879/hb5301Isup2.hkl

Additional supplementary materials:  crystallographic information; 3D view; checkCIF report

## Figures and Tables

**Table:** 

Hg1—N2	2.661 (7)
Hg1—Cl1^i^	2.970 (2)
Hg1—Cl1	2.375 (2)

**Table:** 

Hg1—Cl1—Hg1^ii^	91.31 (7)

Symmetry codes: (i) 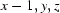; (ii) 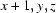.

**Table 2:** Hydrogen-bond geometry (Å, °)

*D*—H⋯*A*	*D*—H	H⋯*A*	*D*⋯*A*	*D*—H⋯*A*
N3—H3*A*⋯O1^iii^	0.86	2.01	2.864 (12)	176
N3—H3*B*⋯N1	0.86	2.40	2.758 (12)	105
N3—H3*B*⋯N1^iv^	0.86	2.54	3.198 (12)	134

Symmetry codes: (iii) 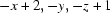; (iv) 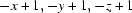.

**Fig. 1.**
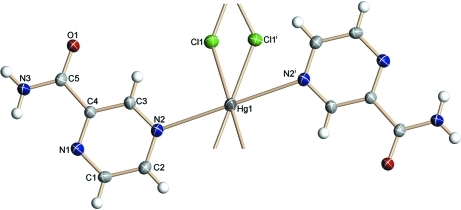
The molecular staucture with displacement ellipsoids drawn at 30% probability level.

**Fig. 2.**
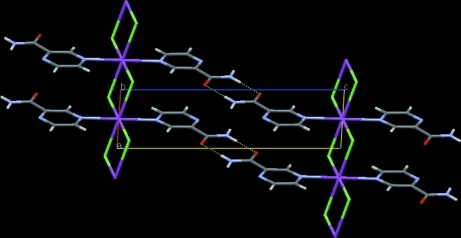
A packing diagram of (I) in b-directrion. Hydrogen bonds are shown as dashed lines.

## supplementary crystallographic information

### Comment

The coordination chemistry of parazineamides is rich. Examples of coordination *via* the pyrazine N atoms, the carbonyl O atoms and the amide N atoms of the ligand in a non-, mono-, or bis-deprotonated form are known (Hausmann and Brooker, 2004; Cati & Stoeckli-Evans, 2004; Miyazaki *et al.* 2007) and metal complexes of the ligands have been used extensively to mimic the properties of biologically active systems. Here we synthesized the title compound, (I), and report here its crystal structure.

The asymmetric unit of the title compound, (I), contains one half-molecule (Fig. 1). The Hg^II^ atom is six-coordinated in a distorted octahedral configuration by two N atoms from pyrazine amides and four bridging Cl atoms. The bridging function of chloro atoms leads to a one-dimensional chain structure. The Hg—Cl and Hg—N bond lengths and angles (Table 1) are within normal ranges. In the crystal structure (Fig. 2), intermolecular N—H···O and N—H···N hydrogen bonds (Table 2) result in the formation of a supramolecular structure, in which they may be effective in the stabilization of the structure.

### Experimental

A solution of pyrazineamide (0.246 g, 2.0 mmol) in methanol (10 ml) was added to a solution of HgCl_2_ (0.272 g, 1.0 mmol) in methanol (5 ml) at room temperature. Colourless plates of (I) were obtained by slow evaporation from methanolic solution after one week (yield; 0.359 g, 69.3%).

### Refinement

All of the H atoms were positioned geometrically with C—H = 0.93 and 0.86Å for aromatic ring and NH_2_ hydrogen atoms respectively, and constrained to ride on their parent atoms, with *U*_iso_(H) = 1.2*U*_eq_(C). The largest peak and deppest hole are near to Hg (0.87 and 0.75Å respectively).

### Crystal data

**Table:**

[HgCl_2_(C_5_H_5_N_3_O)_2_]	*Z* = 1
*M**_r_* = 517.73	*F*(000) = 242
Triclinic, *P*1	*D*_x_ = 2.403 Mg m^−^^3^
Hall symbol: -P 1	Mo *K*α radiation, λ = 0.71073 Å
*a* = 3.8451 (8) Å	Cell parameters from 976 reflections
*b* = 6.4170 (13) Å	θ = 3.3–29.1°
*c* = 14.854 (3) Å	µ = 11.14 mm^−^^1^
α = 101.14 (3)°	*T* = 298 K
β = 92.53 (3)°	Plate, colourless
γ = 94.69 (3)°	0.48 × 0.15 × 0.06 mm
*V* = 357.73 (13) Å^3^	

### Data collection

**Table:**

Stoe IPDS II diffractometer	1880 reflections with *I* > 2σ(*I*)
ω scans	*R*_int_ = 0.096
Absorption correction: numerical [optically, by *X-RED* and *X-SHAPE* (Stoe & Cie, 2005)]	θ_max_ = 29.1°, θ_min_ = 3.3°
*T*_min_ = 0.150, *T*_max_ = 0.515	*h* = −5→4
4201 measured reflections	*k* = −8→8
1887 independent reflections	*l* = −20→20

### Refinement

**Table:**

Refinement on *F*^2^	0 restraints
Least-squares matrix: full	H-atom parameters constrained
*R*[*F*^2^ > 2σ(*F*^2^)] = 0.054	*w* = 1/[σ^2^(*F*_o_^2^) + (0.110*P*)^2^ + 0.204*P*] where *P* = (*F*_o_^2^ + 2*F*_c_^2^)/3
*wR*(*F*^2^) = 0.144	(Δ/σ)_max_ < 0.001
*S* = 1.08	Δρ_max_ = 3.25 e Å^−^^3^
1887 reflections	Δρ_min_ = −3.75 e Å^−^^3^
97 parameters	

### Special details

**Table:**

Geometry. All e.s.d.'s (except the e.s.d. in the dihedral angle between two l.s. planes) are estimated using the full covariance matrix. The cell e.s.d.'s are taken into account individually in the estimation of e.s.d.'s in distances, angles and torsion angles; correlations between e.s.d.'s in cell parameters are only used when they are defined by crystal symmetry. An approximate (isotropic) treatment of cell e.s.d.'s is used for estimating e.s.d.'s involving l.s. planes.

### Fractional atomic coordinates and isotropic or equivalent isotropic displacement parameters (Å^2^)

**Table:**

	*x*	*y*	*z*	*U*_iso_*/*U*_eq_	
C1	0.397 (3)	0.5265 (12)	0.2863 (6)	0.0431 (16)	
H1	0.3077	0.6583	0.3014	0.052*	
C2	0.400 (3)	0.4268 (13)	0.1935 (6)	0.0431 (16)	
H2	0.3177	0.4953	0.1482	0.052*	
C3	0.632 (2)	0.1435 (13)	0.2363 (6)	0.0391 (14)	
H3	0.7083	0.008	0.2215	0.047*	
C4	0.639 (2)	0.2438 (11)	0.3279 (5)	0.0341 (12)	
C5	0.793 (2)	0.1365 (12)	0.3999 (6)	0.0385 (14)	
N1	0.520 (2)	0.4354 (11)	0.3536 (5)	0.0429 (14)	
N2	0.519 (2)	0.2350 (11)	0.1690 (5)	0.0412 (13)	
N3	0.784 (3)	0.2340 (13)	0.4863 (6)	0.0516 (19)	
H3A	0.8724	0.1795	0.5296	0.062*	
H3B	0.6888	0.352	0.4994	0.062*	
O1	0.924 (3)	−0.0327 (12)	0.3755 (5)	0.0539 (18)	
Cl1	0.8689 (6)	−0.2371 (3)	0.05218 (16)	0.0444 (4)	
Hg1	0.5	0	0	0.03963 (18)	

### Atomic displacement parameters (Å^2^)

**Table:**

	*U*^11^	*U*^22^	*U*^33^	*U*^12^	*U*^13^	*U*^23^
C1	0.054 (4)	0.036 (3)	0.041 (4)	0.016 (3)	−0.005 (3)	0.007 (3)
C2	0.056 (4)	0.042 (3)	0.034 (4)	0.011 (3)	0.000 (3)	0.012 (3)
C3	0.046 (4)	0.042 (3)	0.029 (3)	0.016 (3)	0.001 (3)	0.004 (2)
C4	0.038 (3)	0.036 (3)	0.029 (3)	0.009 (2)	−0.001 (3)	0.006 (2)
C5	0.045 (4)	0.040 (3)	0.030 (3)	0.006 (3)	−0.004 (3)	0.008 (2)
N1	0.052 (4)	0.038 (3)	0.038 (3)	0.012 (2)	−0.001 (3)	0.003 (2)
N2	0.049 (4)	0.045 (3)	0.031 (3)	0.014 (2)	−0.001 (3)	0.007 (2)
N3	0.077 (6)	0.045 (3)	0.035 (3)	0.032 (3)	−0.003 (3)	0.003 (3)
O1	0.084 (5)	0.047 (3)	0.033 (3)	0.033 (3)	0.001 (3)	0.004 (2)
Cl1	0.0448 (9)	0.0477 (9)	0.0434 (10)	0.0146 (7)	0.0022 (8)	0.0114 (7)
Hg1	0.0397 (2)	0.0505 (3)	0.0305 (2)	0.01765 (14)	−0.00015 (15)	0.00733 (15)

### Geometric parameters (Å, °)

**Table:**

C1—N1	1.340 (12)	C5—N3	1.318 (11)
C1—C2	1.404 (12)	N3—H3A	0.86
C1—H1	0.93	N3—H3B	0.86
C2—N2	1.338 (11)	Cl1—Hg1^i^	2.970 (2)
C2—H2	0.93	Hg1—Cl1^ii^	2.375 (2)
C3—N2	1.327 (11)	Hg1—N2^ii^	2.661 (7)
C3—C4	1.387 (10)	Hg1—Cl1^iii^	2.970 (2)
C3—H3	0.93	Hg1—N2	2.661 (7)
C4—N1	1.338 (10)	Hg1—Cl1^iv^	2.970 (2)
C4—C5	1.506 (11)	Hg1—Cl1	2.375 (2)
C5—O1	1.232 (11)		
			
N1—C1—C2	121.3 (7)	C5—N3—H3A	120
N1—C1—H1	119.4	C5—N3—H3B	120
C2—C1—H1	119.4	H3A—N3—H3B	120
N2—C2—C1	121.3 (8)	Hg1—Cl1—Hg1^i^	91.31 (7)
N2—C2—H2	119.4	Cl1^ii^—Hg1—Cl1	180.0
C1—C2—H2	119.4	Cl1^ii^—Hg1—N2	89.49 (17)
N2—C3—C4	122.0 (7)	Cl1—Hg1—N2	90.51 (17)
N2—C3—H3	119	Cl1^ii^—Hg1—N2^ii^	90.51 (17)
C4—C3—H3	119	Cl1—Hg1—N2^ii^	89.49 (17)
N1—C4—C3	121.7 (8)	N2—Hg1—N2^ii^	180.0
N1—C4—C5	119.3 (7)	Cl1^ii^—Hg1—Cl1^iii^	91.31 (7)
C3—C4—C5	118.9 (7)	Cl1—Hg1—Cl1^iii^	88.69 (7)
O1—C5—N3	124.0 (8)	N2—Hg1—Cl1^iii^	94.05 (18)
O1—C5—C4	119.1 (7)	N2^ii^—Hg1—Cl1^iii^	85.95 (18)
N3—C5—C4	116.9 (7)	Cl1^ii^—Hg1—Cl1^iv^	88.69 (7)
C4—N1—C1	116.7 (7)	Cl1—Hg1—Cl1^iv^	91.31 (7)
C3—N2—C2	117.0 (7)	N2—Hg1—Cl1^iv^	85.95 (18)
C3—N2—Hg1	116.0 (5)	N2^ii^—Hg1—Cl1^iv^	94.05 (18)
C2—N2—Hg1	126.8 (6)	Cl1^iii^—Hg1—Cl1^iv^	180.0
			
N1—C1—C2—N2	1.5 (15)	C1—C2—N2—Hg1	174.2 (7)
N2—C3—C4—N1	2.4 (13)	Hg1^i^—Cl1—Hg1—N2	−94.04 (18)
N2—C3—C4—C5	−175.8 (8)	Hg1^i^—Cl1—Hg1—N2^ii^	85.96 (18)
N1—C4—C5—O1	−174.9 (9)	Hg1^i^—Cl1—Hg1—Cl1^iii^	0
C3—C4—C5—O1	3.3 (12)	Hg1^i^—Cl1—Hg1—Cl1^iv^	180
N1—C4—C5—N3	4.0 (12)	C3—N2—Hg1—Cl1^ii^	163.8 (6)
C3—C4—C5—N3	−177.8 (9)	C2—N2—Hg1—Cl1^ii^	−10.0 (8)
C3—C4—N1—C1	−0.5 (12)	C3—N2—Hg1—Cl1	−16.2 (6)
C5—C4—N1—C1	177.7 (8)	C2—N2—Hg1—Cl1	170.0 (8)
C2—C1—N1—C4	−1.4 (13)	C3—N2—Hg1—Cl1^iii^	−104.9 (6)
C4—C3—N2—C2	−2.3 (13)	C2—N2—Hg1—Cl1^iii^	81.3 (8)
C4—C3—N2—Hg1	−176.7 (6)	C3—N2—Hg1—Cl1^iv^	75.1 (6)
C1—C2—N2—C3	0.4 (13)	C2—N2—Hg1—Cl1^iv^	−98.7 (8)

Symmetry codes: (i) *x*+1, *y*, *z*; (ii) −*x*+1, −*y*, −*z*; (iii) −*x*+2, −*y*, −*z*; (iv) *x*−1, *y*, *z*.

### Hydrogen-bond geometry (Å, °)

**Table:**

*D*—H···*A*	*D*—H	H···*A*	*D*···*A*	*D*—H···*A*
N3—H3A···O1^v^	0.86	2.01	2.864 (12)	176
N3—H3B···N1	0.86	2.40	2.758 (12)	105
N3—H3B···N1^vi^	0.86	2.54	3.198 (12)	134

Symmetry codes: (v) −*x*+2, −*y*, −*z*+1; (vi) −*x*+1, −*y*+1, −*z*+1.

## References

[bb2] Cati, D. S. & Stoeckli-Evans, H. (2004). *Acta Cryst.* E**60**, m177–m179.

[bb3] Farrugia, L. J. (1997). *J. Appl. Cryst.* **30**, 565.

[bb4] Farrugia, L. J. (1999). *J. Appl. Cryst.* **32**, 837–838.

[bb5] Hausmann, J. & Brooker, S. (2004). *Chem. Commun.* pp. 1530–1531. 10.1039/b403905j 15216365

[bb8] Mir Mohammad Sadegh, B., Azhdari Tehrani, A. & Khavasi, H. R. (2010). *Acta Cryst.* E**66**, m158. 10.1107/S1600536810001182 PMC2979956 21579633

[bb6] Miyazaki, S., Ohkubo, K., Kojima, T. & Fukuzumi, S. (2007). *Angew. Chem. Int. Ed.* **46**, 905–908. 10.1002/anie.200604028 17200969

[bb7] Sheldrick, G. M. (2008). *Acta Cryst.* A**64**, 112–122. 10.1107/S0108767307043930 18156677

[bb1] Stoe & Cie (2005). *X-AREA*, *X-RED* and *XSHAPE* Stoe & Cie, Darmstadt, Germany.

